# AI based monitoring violent action detection data for in-vehicle scenarios

**DOI:** 10.1016/j.dib.2022.108564

**Published:** 2022-08-31

**Authors:** Nelson R.P. Rodrigues, Nuno M.C. da Costa, Rita Novais, Jaime Fonseca, Paulo Cardoso, João Borges

**Affiliations:** a Algoritmi Center, University of Minho, Guimarães, Portugal; b Polytechnic Institute of Cavado and Ave, Barcelos, Portugal

**Keywords:** Action recognition, Autonomous vehicles, Deep learning, Violent action, Dataset

## Abstract

With the evolution of technology associated with mobility and autonomy, Shared Autonomous Vehicles will be a reality. To ensure passenger safety, there is a need to create a monitoring system inside the vehicle capable of recognizing human actions. We introduce two datasets to train human action recognition inside the vehicle, focusing on violence detection. The InCar dataset tackles violent actions for in-car background which give us more realistic data. The InVicon dataset although doesn't have the realistic background as the InCar dataset can provide skeleton (3D body joints) data. This datasets were recorded with RGB, Depth, Thermal, Event-based, and Skeleton data. The resulting dataset contains 6 400 video samples and more than 3 million frames, collected from sixteen distinct subjects. The dataset contains 58 action classes, including violent and neutral (i.e., non-violent) activities.

## Specifications Table

**Table:** 

Subject	Computer Science, Computer Vision and Pattern Recognition
Specific subject area	Image Processing, Violent Action Recognition
Type of data	Images Skeleton Annotations
How the data were acquired	Images were acquired using, Microsoft Kinect Azure, FLIR ADK and CeleX-5-MIPI, connected to a machine using their respective sdk to capture the frames at a frequency of 30 Hz. Body pose data (skeleton) are recorded through the Vicon Suit on the Vicon system; global positioning data are recorded with Vicon Shogun through the creation of a 3d skeleton subjects at a frequency of 120 Hz.
Data format	RAW
Description of data collection	The binary files contain the raw data. Then the raw data was converted to images. Due to the different sensors, temporal synchronization was required. Considering a constant frame rate and initial time delay, from each sensor, the calibration was focused in establishing a global timestamp for each sensor sample and removing the initial time delay. Timestamps were provided through the recording system real-time clock (RTC), while the initial time delay was extracted from each sensor number of samples in buffer divided by the frame rate.
Data source location	Guimarães, Portugal, University of Minho, Algoritmi Center, Latitude: 41.450715, Longitude: -8.293490
Data accessibility	Repository name: datarepositorium Direct URL to InCar data: https://doi.org/10.34622/datarepositorium/1S8QVP Direct URL to InVicon data: https://doi.org/10.34622/datarepositorium/WWUTUT
Related research article	João Borges, Bruno Oliveira, Helena Torres, Nelson Rodrigues, Sandro Queirós, Maximilian Shiller, Victor Coelho, Johannes Pallauf, José Mendes, Jaime Fonseca, **Automated Generation of Synthetic in-Car Dataset for Human Body Pose Detection**[1] doi:10.5220/0009316205500557 João Borges, Sandro Queirós, Bruno Oliveira, Helena Torres, Nelson Rodrigues, Victor Coelho, Johannes Pallauf, José Henrique Brito, José Mendes, Jaime C. Fonseca, **A system for the generation of in-car human body pose datasets**[2] doi:10.1007/s00138-020-01131-z

## Value of the Data

• The data is valuable for the field of computer vision, especially for the tasks of monitoring the behavior of passengers for shared autonomous vehicles scenarios, where the driver can't ensure the security of the vehicle, and the passengers between themselves.
• The data provided can be used to train Deep Learning models to recognize the actions in the dataset using several features (RGB, Depth, Thermal, Event-based, and Skeleton) or all at the same time. It is also useful as a reference dataset for benchmarking models.
• This dataset comprehends 58 classes of actions, including violent and neutral, in the form of both RGB, Depth, Thermal, Event-based images, and Skeleton, temporally and spatially (for InVicon Dataset) aligned. Which provides lots of data to explore and combine different features for further insights and development experiments.

## 1 Data Description

In recent years, car manufacturers and software companies have made significant investments in developing the concept of autonomous driving. With the evolution of this area, Shared Autonomous Vehicles (SAV) are expected to be the next revolution in transportation systems, bringing higher levels of automation and the need to implement intelligent services in vehicles to ensure the well-being and safety of passengers in shared ride.

Also, from the passenger's perspective, it will be a paradigm shift as the driver becomes obsolete and passengers are the only human agent in the vehicle. This way, SAV concepts must be carefully thought out to guarantee the safety of passengers and the vehicle. In this context, the need to monitor the environment inside the car will become crucial, namely, passenger monitoring, because it can detect violent actions that may arise between passengers (passenger-passenger interaction), increasing their confidence in the use of SAV. On the other hand, it will be possible to identify any passenger action that jeopardizes the vehicle's integrity (passenger-vehicle interaction), safeguarding the company's interests that provides the service.

The previous work developed on [1] consists of a toolchain for the generation of realistic synthetic for human body pose detection in an in-car environment. This toolchain demonstrated the potential for increased algorithm accuracy during body pose estimation, although the toolchain could not give the data realism that is so important for real use cases. To overcome the issue of realism background we present the InCar Dataset, which gives us 58 violent actions inside the vehicle, with RGB, Thermal, and Event-based features.

Although the InCar Dataset have realistic frames, couldn't provide 3d body joints due to occlusion inside the vehicle. To avoid this problem, we developed a novel system in [2] for the generation of datasets of human body poses in cars. The system was demonstrated to be able to generate datasets through a specific setup consisting of an inertial suit, a global positioning system, and a ToF camera, coupled with a set of calibration procedures. Although this solves the issue of having realist images, and still having the body pose information, there still have some limitations mostly coming from the inertial suit.

So, to overcome the issue previously mentioned in [2], we created a system that combines a computer vision sensor, a motion capture suit, and the Vicon system. The computer vision sensor provides image data; the motion sensor provides 3d skeleton, and the Vicon system provides the global positioning inside the scenario. The output data are comprised of human body poses given concerning the camera's coordinate system in 2D and 3D for the computer vision sensor frame (RGB, IR, depth, and point-cloud). With this system we created the InVicon dataset. This dataset has the same number of violent actions as the InCar dataset but enhance the skeleton data for different features.

### 1.1 Subjects Characterization

**Fig. 1:**
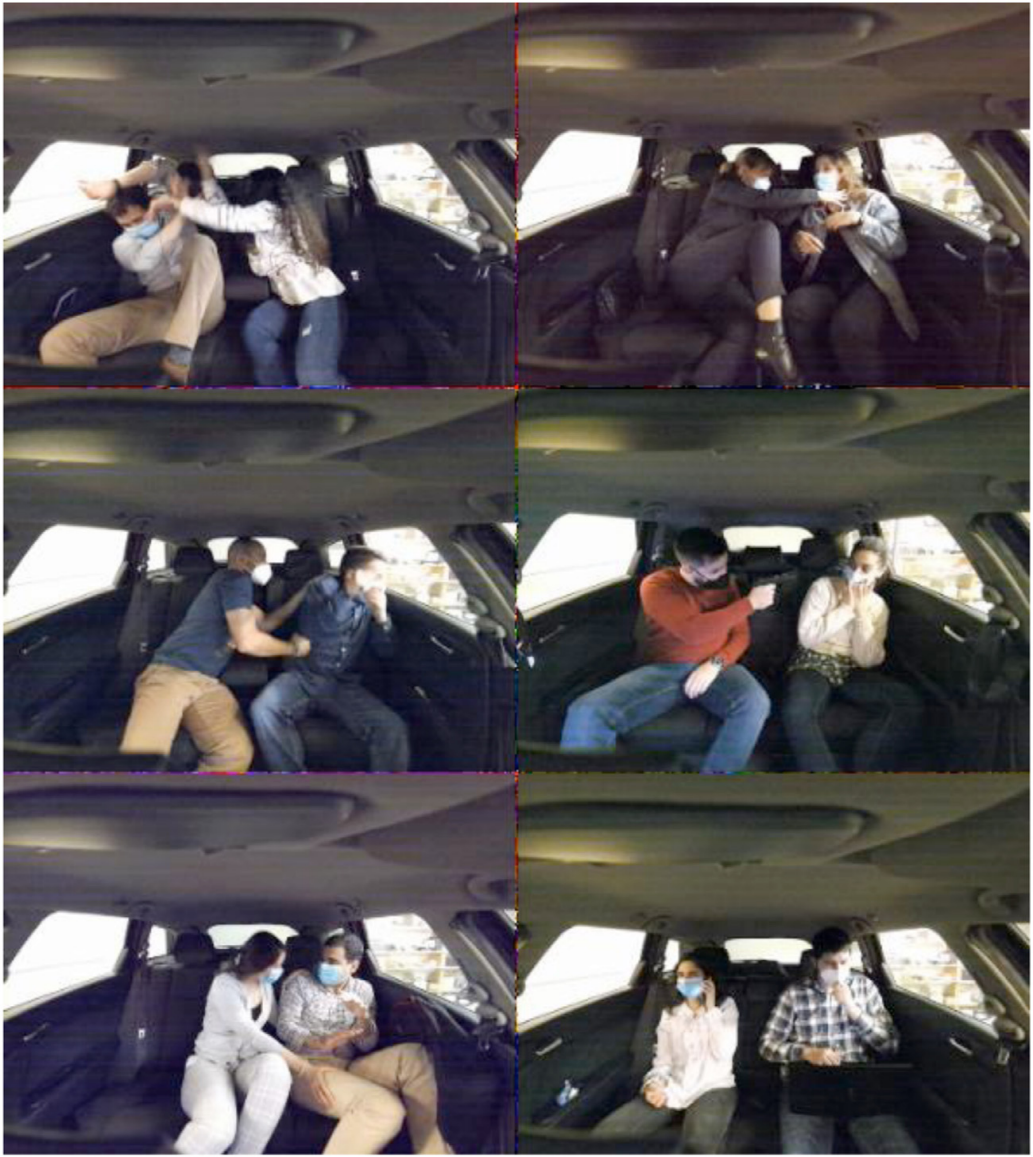
Sample frames from the proposed MoLa InCar (from top left to bottom right, actions are slapping, kicking, punching, pointing a gun, sexual harassment, and independent action, in this case, the left passenger is on the cellphone and the right in the laptop).

For the dataset recording, 16 subjects were invited (9 males and 7 females). These subjects are from 3 different countries (i.e., Portuguese, Brazilian, and Iranian), with ages ranging from 23 and 40, height between 1.6 m and 1.9 m. Fig. 1 shows the variety of subjects in age, gender and height. Each subject is assigned a consistent ID number over the entire dataset. To allow interaction, recordings were always made with two subjects, while the 16 subjects were divided into four different groups of 4 subjects, thus creating sequences where subjects never interacted. For each group (A-D) presented in Table 1, four different pairs of records were created.

**Table 1:** Groups of subjects.

Subjects ID	Group
1,2,3,4	A
5,6,7,8	B
9,10,11,12	C
13,14,15,16	D

### 1.2 Action Classes

A total of twenty action scenarios were created, divided into two major categories, with twelve violent actions sequences (see Table 2) and eight non-violent actions sequences (see Table 3). Moreover, each action sequence typically has between 3 to 4 individual and interactive actions, thus totaling 58 different actions for the entire dataset. For each category there are actions with and without objects (violent and non-violent).

**Table 2:** Violent scenarios.

Scenario	Description
Human-Human Interaction Without Objects	

C1	Discuss (P1,P2) – Pushing (P1) – Punching (P1).
C2	Singing/dancing (P1) – Asking for a kiss (P1) – Refusing kiss (P2) – Slapping (P1) – Pulling/Pushing (P1)
C3	Arguing (P1,P2) – Showing middle finger (P2) – Kicking (P1) – Strangling (P1)
C4	Arguing (P1,P2) – Threatening to hit (P2) – Provoking (P1) – Slapping (P1)

Human-human interaction with objects	

C5	Sexual Harassment: Approaching/Coming closer to the other person (P1) – Stroking the hair (P1) – Touching on the body (P1)- Beating with backpack/purse (P2)
C6	Greeting/complimenting (P1,P2) – Showing something on phone (P1) – Threatening with scissors (P1) – Stealing/asking for the wallet (P1)
C7	Arguing (P1,P2) – Picking a gun from backpack/purse/clothes (P1) – Pointing a gun (P1) – Punching with the gun (P1)
C8	Arguing (P1,P2)- Picking knife from backpack/purse/clothes (P1) - Pointing knife (P1) Stabbing (P1)
C9	Coming closer to the other person (P1) - Threatening with knife (P1) – Touching on the body (P1)
C10	Playing on the phone (P1) – Playing on the phone (P2)- Slap (s) (P1) - Holding and punching (P2)
C11	Sleeping (P2) - Drinking (P1) - Throwing bottle (P1) - Pushing (P2)
C12	Playing on the phone (P2) - Moving closer/ looking at the cell phone (P1) - Pulling away (P2)- Pulling/Pushing (P1)

**Table 3:** Non-violent scenarios.

Scenario	Description	
Human-Human Interaction Without Objects		

C13	Talking/discussing (P1,P2) - P2 begins to cry - Hugging (P1,P2)	
C14	P1 asks P2 to take pictures - P2 takes pictures - P2 shows P1 what the pictures look like	

Independent actions of the subjects		

	P1 – Person	P2 – Person 2
C15	Applying lipstick - Fixing hair	Sleeping
C16	Sneezing (1x or +) – Picking tissue from purse, bag, clothes - Wiping nose	Read the book
C17	Yawn - Stretching, Twisting, or Stretching of the neck	Putting headphones - listening to music - singing / dancing
C18	Eating - Drinking	Taking pictures on phone
C19	Answering call/talking	Doing stuff on pc - Coughing
C20	Writing on the notepad	Applying alcohol gel

### 1.3 Annotations

This dataset contains 6 400 video samples, and more than 3 million frames, which was collected using 58 action classes, from 16 subjects (different ages, gender, and height). Although our labeling is primary binary for the moment (violent and non-violent). We also provided information related to the clothing color, material, and skin tone, according to the Fitzpatrick scale [3] of each subject for each recording.

**Fig. 2:**
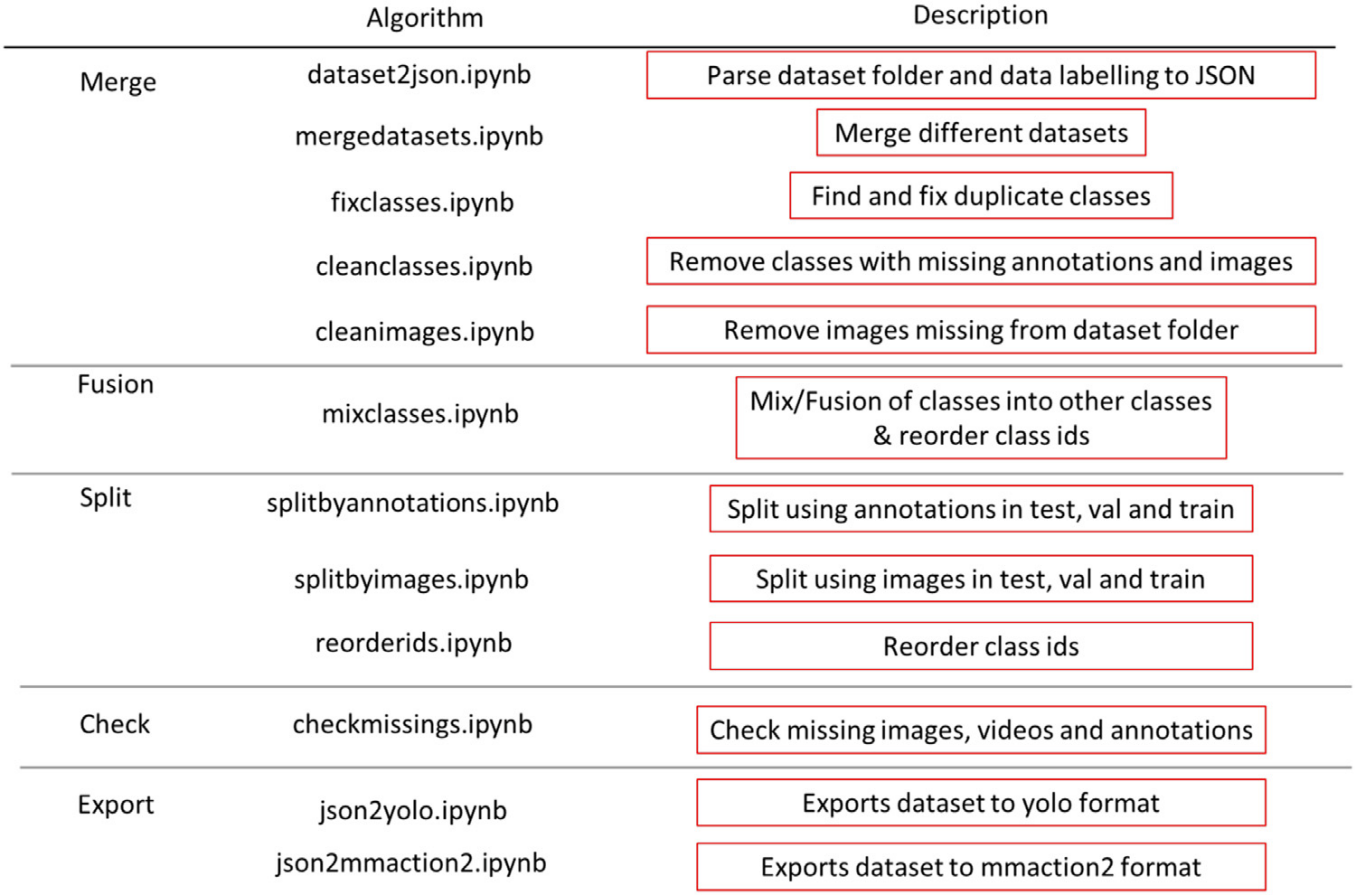
Annotation pipeline. The annotation pipeline consists of 5 types of algorithms: merge, fusion, split, check and export.

For the annotations we used the MoLAnnotate Toolkit [4]. This pipeline includes (see Fig. 2) first a merge subroutine, where raw or public datasets data are parsed to our JSON unified format: the conversion of dataset directory and raw labelling to JSON, the “dataset2json.ipynb”; merging JSON annotations of one or more datasets (public or/and custom), the “mergedatasets.ipynb”; fix classes with duplicate names “fixclasses.ipynb”; clean classes with missing annotations and images “cleanclasses.ipynb” and check missing images and remove respective annotations “cleanimages.ipynb”.

A fusion algorithm that mix classes into other classes and reorders the ids, the “mixclasses.ipynb”.

A split subroutine, where one can split by annotations “splitbyannotations.ipynb” (recommended to have an equilibrium between classes) or split by images “splitbyimages.ipynb” and reorder ids if needed “reorderids.ipynb”.

A check pipeline was also implemented to check missing images, videos and annotations “checkmissings.ipynb”.

Finally, an export subroutine, where different export methods are added as the examples on Fig. 2 “json2yolo.ipynb” and “json2mmaction2.ipynb”.

Compared to the current datasets for this task, our dataset is more extensive and contains much more variety. Additionally, a more specific violent action labelling is necessary to improve the introduced dataset.

## 2 Experimental Design, Materials and Methods

### 2.1 Collection Setup

**Fig. 3:**
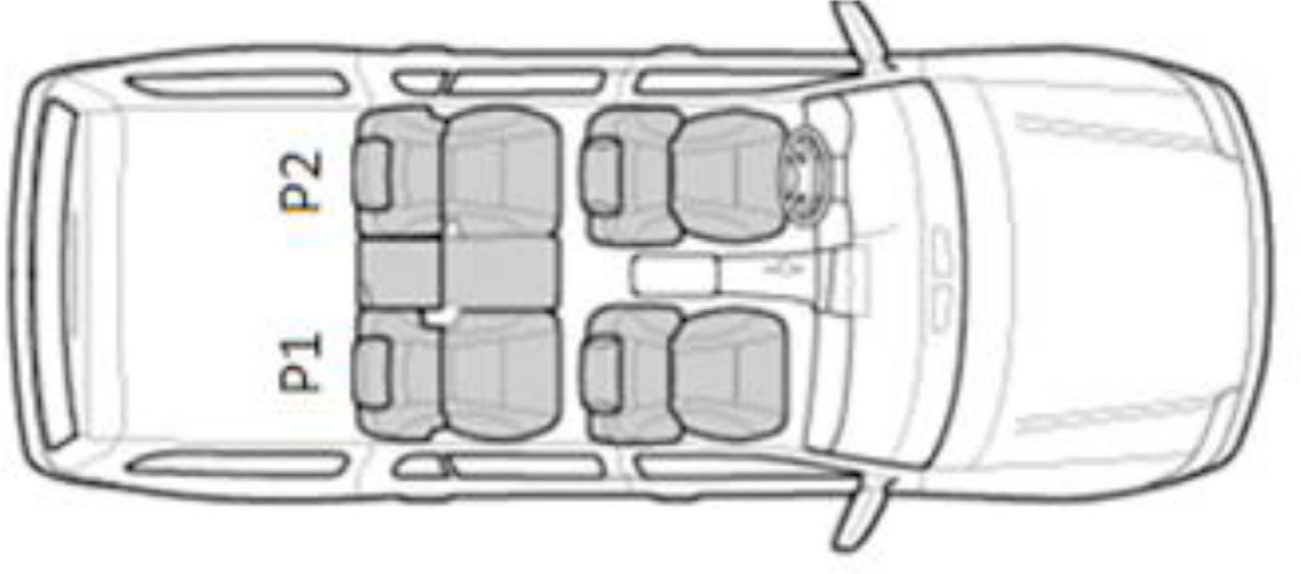
Position of the subjects in the records.

All recordings for the InCar dataset were made inside a vehicle testbed, that is, Fig. 3, with sensors placed in the rear-view mirror position and subjects in the back seats (i.e., P1 and P2). Each action sequence was recorded for each record number, with extra subject seat rotation (i.e., subjects change seats and repeat action sequence recording) and redundancy (i.e., 2 recordings for each action sequence). For the violent scenarios (C1-C12), the recording time was twenty seconds, and for the nonviolent scenarios (C13-C20), the recording time was fifteen seconds. Due to the recordings that were performed inside the lab, the lighting conditions are adapted to that environment.

We decided to use two subjects for the actions scenarios, due to the interactions of the type of violent actions recorded, if we consider the use of more than two subjects it may have a lot more of occlusions.

For the InVicon dataset the procedure for the recording scenarios was the same but outside the vehicle due to occlusions.

### 2.2 Data-Modalities

To collect the datasets, four sensors were used:

(1) The Microsoft Kinect Azure sensor from which two primary data modalities were captured, i.e., RGB frames, depth maps and point-cloud. The RGB frames are recorded with a resolution of 2048 × 1536. Depth maps are sequences of two-dimensional depth values in millimeters, with a resolution of 640 × 576. Point-cloud is comprised by the 3D position of each pixel in the depth map, i.e., voxels. Furthermore, all samples were recorded at 30 frames per second (FPS) with a field of view (FoV) of 75° × 65°.
(2) FLIR ADK sensor from which thermal frames were collected, with a resolution of 640 × 512, horizontal FoV of 75°, at 30 FPS.
(3) CeleX-5-MIPI from which events were collected with a resolution of 800 × 1280, at 70MHz. In event intensity mode, the CeleX-5 Sensor detects light intensity change and outputs row/col address of the detected events, the pixel intensity information sampled at the instant when the event is generated, and the off-pixel timestamp when the event is output. With these events collected by the sensor, it is possible, for a period, to create an image, through a specific event frame time, that could be specified from a range between 100us to 100ms, being able to create different types of event image frames (such as event gray pic, event accumulated gray pic, etc.).
(4) The Vicon system from which is captures the motion capture to extract the skeleton data. The system works at 120Hz with a resolution of 1280 × 1024.

**Fig. 4:**
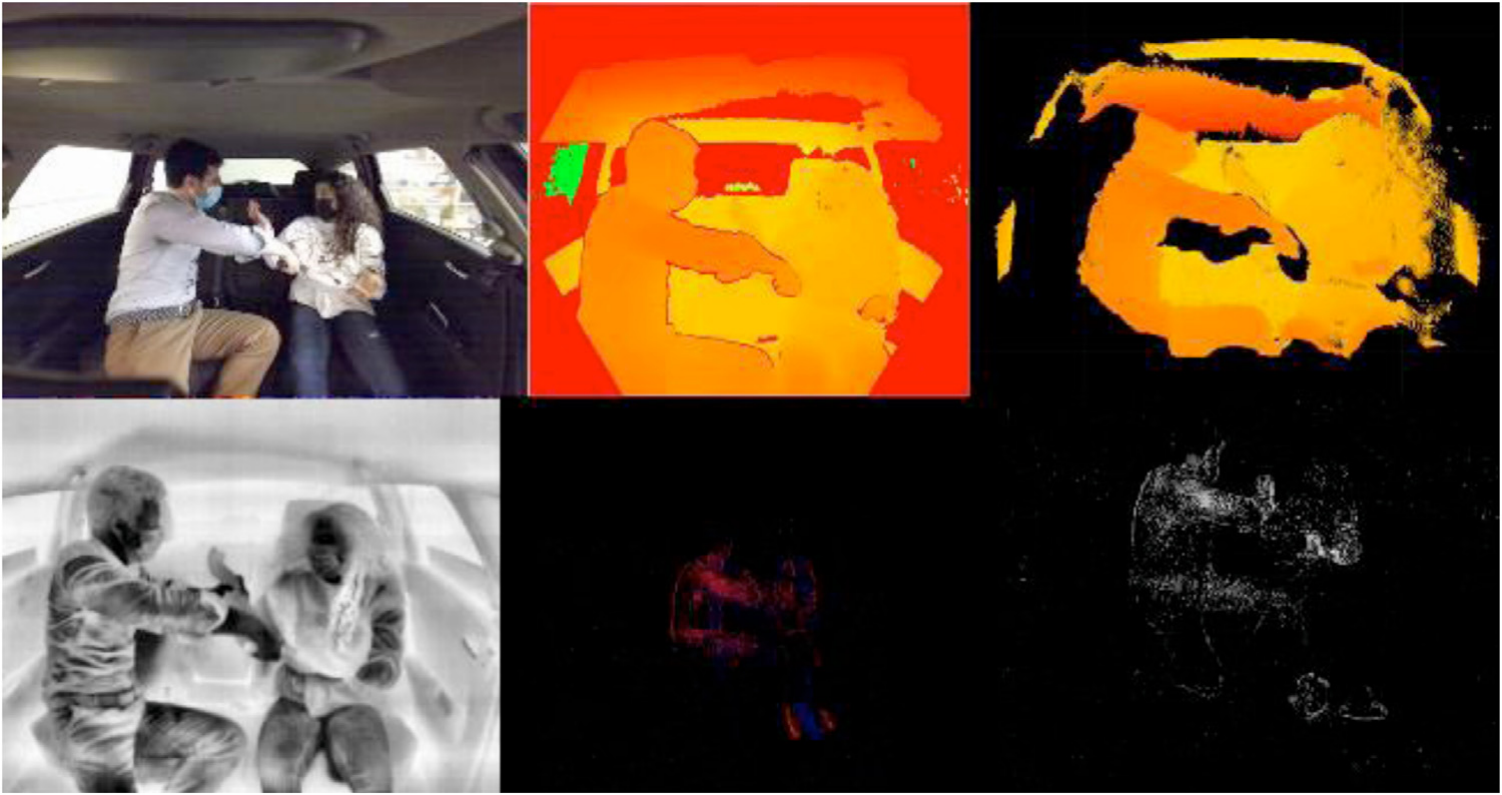
From Top Left to Bottom Right: RGB, Depth, Point-cloud, Thermal, NVS, and Events Grayscale).

Fig. 4 presents an example of violent action (pointing knife) with different features from the first three sensors mentioned above.

### 2.3 Temporal Alignment

To temporally align the Kinect camera with the Vicon system, we create a setup where the Vicon system gives the trigger to the Kinect, and in this case, we used the 3.5 mm sync in input from the Kinect device. So, from the moment we start recording, the Vicon system sends a signal to the Kinect device and waits until the Kinect is ready to capture.

Due to the different sensors, temporal synchronization was required. Considering a constant frame rate and initial time delay, from each sensor, the calibration was focused in establishing a global timestamp for each sensor sample and removing the initial time delay. Timestamps were provided through the recording system real-time clock (RTC), while the initial time delay was extracted from each sensor number of samples in buffer divided by the frame rate.

### 2.4 Spatially Alignment

To develop a system capable of generating RGB images with associated human body pose ground-truth, several systems are required:

• A Kinect sensor for image capture (K being the position of the camera's optical center).
• A global object positioning system, such as the Vicon system (W being the Vicon's global coordinate system).
• A Vicon suit capable of capturing human body pose ground-truth (J being the body joints).

**Fig. 5:**
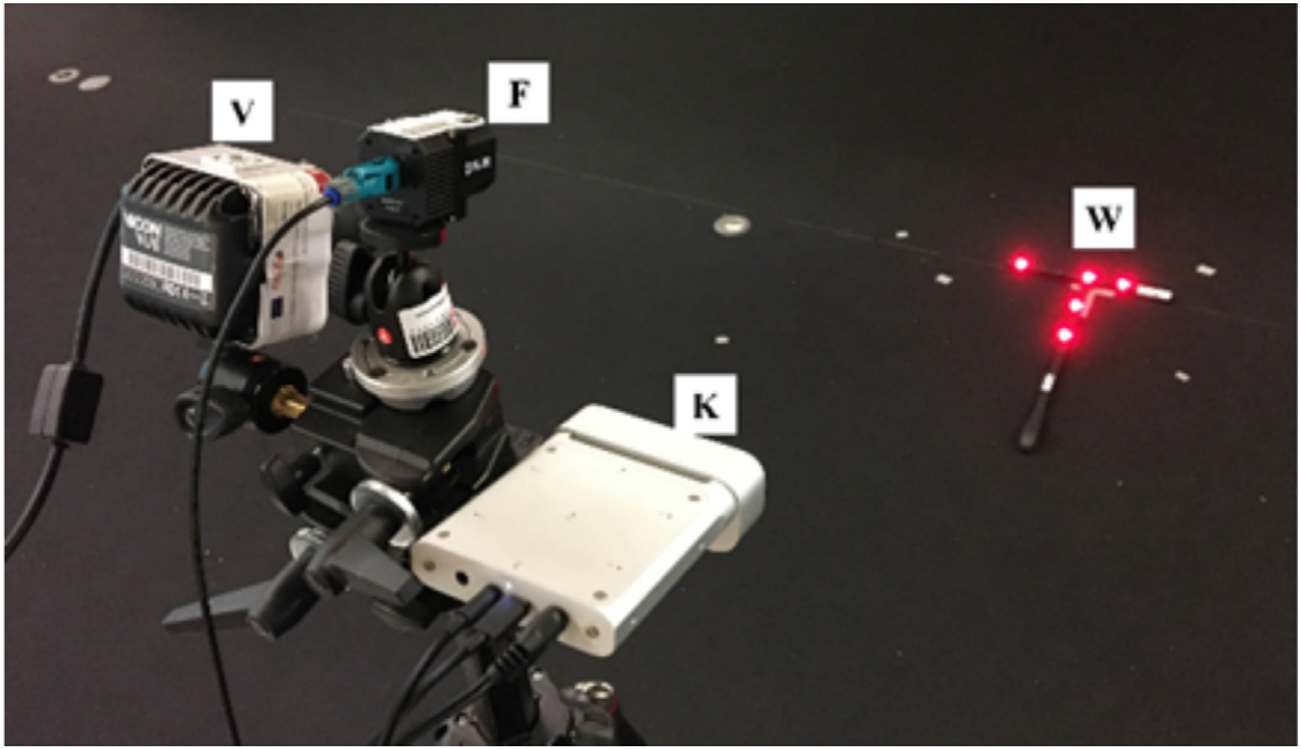
3D representation of coordinate systems when recording the dataset. W: Vicon global coordinate system; K: Kinect optical center.

As Fig. 5 illustrates, there is a certain complexity with the added systems. With it, there is a need to spatially and temporally align the data, to correctly project the human body pose information into the Kinect camera's perspectives.

Considering the goal of generating human body pose ground-truth projected into the Kinect sensor, a spatial calibration is required. In this regard, there was the need to align the Vicon System with the Kinect.

To calibrate the Kinect, we utilize a checkerboard as a calibrate object, and to validate the calibration of the sensor we create an application in MATLAB to see the transformation matrixes to project the 3D Vicon world coordinates into the 2D world of Kinect.

Vicon suit to Vicon World ($T_{O}^{W}$). The ($T_{O}^{W}$) transformation is automatically recorded through the Vicon system and requires the setup of the subjects.

**Fig. 6:**
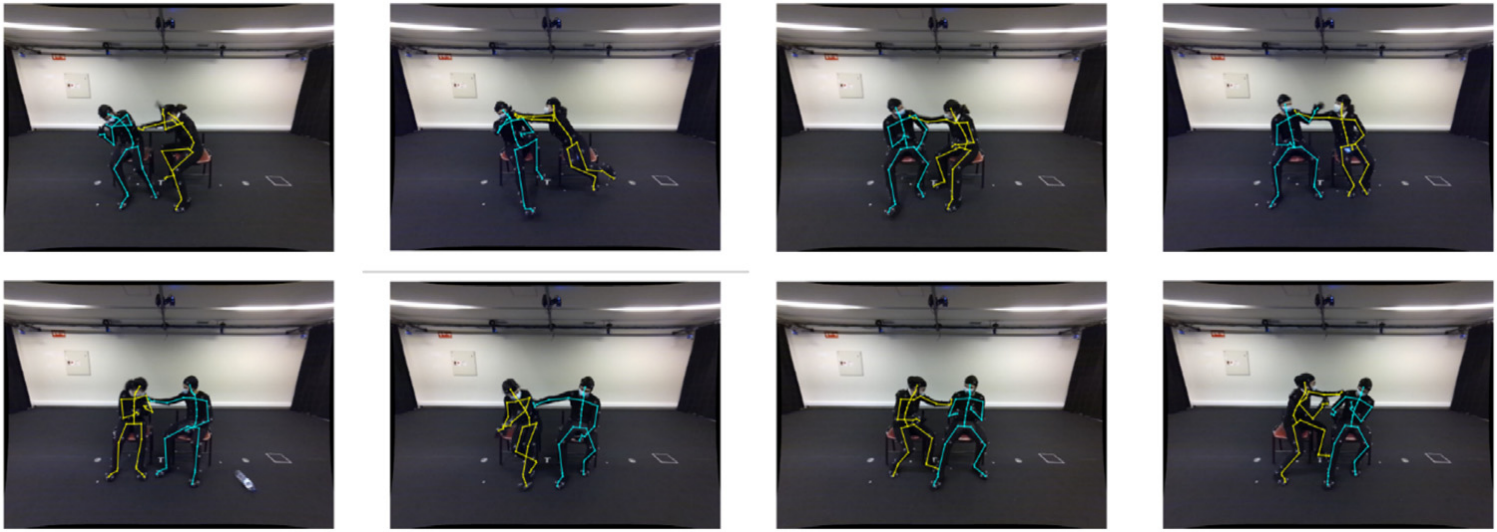
Vicon skeleton projected on RGB frame.

Before we start the collection of the data of each pair of subjects, we needed to calibrate all the sensors. To calibrate the cameras of the Vicon Vero system we followed the normal procedure recommended by the manufactures of the software. The calibrate device is a wand that gives us the coordinate axis of the 3D world of the Vicon system. The Vicon Vue camera was calibrated too in this process. It is important to note that the Vicon Vue Camera is only used in this step for us to be able to project the Vicon's marker 3D coordinates into the camera's image frames (See Fig. 6).

## 3 Conclusions

This study proposes two datasets. The MoLa InCar AR, that includes the violent and non-violent actions inside the vehicle and provides analytical experimental results. The dataset contains a new large-scale action recognition dataset containing 1,280 videos samples per feature, which was collected using 58 action classes, from 16 subjects (different ages, gender, and height). Compared to the current datasets for this task, our dataset is more extensive and contains much more variety. This dataset is expected to be useful for research on action recognition for vehicle scenarios. The MoLa InVicon AR dataset follows the same approach as the previous mentioned dataset, with the same action classes, and the same subjects, but provides skeleton data which could be very useful for action recognition tasks. We consider that these two datasets complement each other by providing lots of features for the researchers explores this problematic.

Additionally, a more specific violent action labelling is necessary to improve the introduced datasets. Furthermore, more features should be studied. These additional dataset features will be released soon.

## Ethics Statements

Informed consent has been obtained from each subject participating in the study, following ethics guidelines, respecting the principle of data minimization and in compliance with the provisions present in the General Data Protection Regulation 2016/679 (EU).

Each participant in this project is voluntary and there is no implicit coercion to participate.

Each participation involves the collection of the personal data mentioned above in the article.

Each contribution to the activity described can be collected by the different capture systems and the data can be processed for the purposes set out.

Each participant is also aware that the image captured where he/she carry out a set of actions will be permanently preserved and can be shared with other scientific communities worldwide.

## CRediT authorship contribution statement

**Nelson R.P. Rodrigues:** Conceptualization, Methodology, Software, Validation, Data curation, Writing – original draft, Visualization, Investigation, Writing – review & editing. **Nuno M.C. da Costa:** Supervision, Writing – review & editing. **Rita Novais:** Conceptualization, Data curation, Writing – original draft, Investigation. **Jaime Fonseca:** Supervision, Writing – review & editing. **Paulo Cardoso:** Supervision, Writing – review & editing. **João Borges:** Supervision, Writing – review & editing.

## Declaration of Competing Interest

The authors declare that they have no known competing financial interests or personal relationships that could have appeared to influence the work reported in this paper.

## Data Availability

MoLa InVicon AR: Dataset for Action Recognition (Original data) (datarepositorium). MoLa InCar AR: Dataset for Action Recognition (Original data) (datarepositorium).
